# β-Glycosphingolipids as Mediators of Both Inflammation and Immune Tolerance: A Manifestation of Randomness in Biological Systems

**DOI:** 10.3389/fimmu.2019.01143

**Published:** 2019-05-22

**Authors:** Yaron Ilan

**Affiliations:** Gastroenterology and Liver Units, Department of Medicine, Hadassah-Hebrew University Medical Center, Jerusalem, Israel

**Keywords:** NKT cells, Gaucher disease, glycosphingolipids, randomness, variability, biological systems

## Abstract

Plasticity in biological systems is attributed to the combination of multiple parameters which determine function. These include genotypic, phenotypic, and environmental factors. While biological processes can be viewed as ordered and sequential, biological randomness was suggested to underline part of them. The present review looks into the concept of randomness in biological systems by exploring the glycosphingolipids-NKT cells example. NKT cells are a unique subset of regulatory lymphocytes which play a role in both inflammation and tolerance. Glycosphingolipids promote an immune balance by changing different arms of the immune system in opposing environments. Traditional immunology looks at skewing the immune system into different directions by different types of activation of the same cell stimulation of different cells subsets, use of different ligands, or different the effect of different immune environments. While these may explain some of the effects, the lack of consistency and opposing results under similar settings may involve randomness which may also be part of real life effects of immunomodulatory agents. It means that several of the biological processes, cannot be explained by simple linear models, and may involve more complex concepts. The application for these concepts for improving therapies to patients with Gaucher disease are discussed.

**SUMMARY**

The use of different ligands that target a variety of cell subsets in different immune environments may underlie differences in the functionality of NKT cells and their variability in response to NKT-based therapies. The novel concept of randomness in biology means that several biological processes cannot be solely explained by simple linear models and may instead involve much more complicated schemes of random disorder. These may have implications on future design of therapeutic regimens for improving the response to current treatments.

## Introduction

Plasticity in biological systems is attributed to a combination of multiple parameters that affect function. It can be simplified by analyzing different subsets of cells with varying functional roles under different conditions. Genotypic, phenotypic, and environmental factors contribute to these differences. While biological processes can be viewed under most settings as fully ordered and sequential, in some cases, biological randomness may underline them. This review evaluates the concept of randomness in biological systems by exploring the example of glycosphingolipids- natural killer T (NKT) cells.

## NKT Cells are a Unique Subset of Regulatory Lymphocytes

NKT cells are a subpopulation of T lymphocytes which exhibits a several immune effector and regulatory functions (1, 2). NKT cells use an invariant T-cell receptor (TCR) alpha chain complexed with a limited repertoire of TCR beta chains to recognize specific lipid antigens (3). A variety of foreign and self-derived glycolipid antigens presented by an MHC class I-related antigen-presenting molecule, CD1d can activated these lymphocytes. The interplay between subsets of NKT cells and other innate and adaptive lymphocytes have significant therapeutic implications for inflammatory and autoimmune diseases, microbial immunity, and cancer (4). Invariant NKT cells (iNKT cells), type I NKT, are a subclass of NKT cells which are at the interface between the innate and adaptive immune systems. They are important mediators of immune responses and were suggested as potential targets for immunotherapy (5). In response to a wide range of antigens, these cells are rapidly activated and exhibit both pro-inflammatory and immunoregulatory characteristics, resulting in harmful or protective roles in infections, autoimmune diseases, allergies, and cancer (6).

CD1d-restricted NKT cells are classified into two subsets. Type I NKT cells express invariant TCRs and react with lipid antigens, including the marine sponge-derived glycolipid alpha-galactosylceramide (α-GalCer). CD1d-restricted type I NKT cells usually act as pro-inflammatory cells but sometimes behave as anti-inflammatory cells. Type II NKT cells react to self- and non-self-lipid ligands and share some properties with both iNKT and conventional T cells (7). They recognize a wide variety of glycolipids, phospholipids, and endogenous hydrophobic peptides via their diverse TCRs. The majorities of CD1d-restricted lipid-reactive human T cells are type II NKT cells (7). CD1d-restricted type II NKT cells function as anti-inflammatory or pro-inflammatory cells depending on the environmental stimulation. α-GalCer-reactive NKT cells that use non-canonical alpha beta TCRs and CD1d-restricted T cells that use gammadelta or delta/alpha beta TCRs have been characterized revealing further diversity among CD1d-restricted T cells (8). Both type I and type II NKT cells react to self and non-self lipids and both types can also be activated by inflammatory cytokines, without the help of antigens (8, 9).

## The Role of NKT Cells in Inflammation

iNKT cells exert regulatory or direct cytotoxic roles to protect the host against infections (3, 10). Microbial pathogens stimulate them either directly via glycolipids or indirectly by inducing cytokine production in antigen-presenting cells (APCs) (11). Organisms lacking cognate CD1d-binding glycolipid antigens are recognized by the semi-invariant TCR of iNKT cells. The bacterial and parasitic cell wall is enriched in glycolipids and lipoproteins, some of which may serve as natural ligands of iNKT cells. These lymphocytes are also activated in response to microbial products including bacterial lipopolysaccharide and by interleukin-12. As an example, during parasite infections, crosstalk occurs between iNKT cells and Th1, Th2, Th17, regulatory T cells (Treg), and innate lymphoid cells. NKT cells can be directly activated by *Leishmania* glycolipids presented by CD1d molecules on APCs, leading to the secretion of diverse cytokines. They can also be activated by an indirect pathway (12). The response of NKT cells in *Leishmania* infections is variable and depends on the infection site, number of parasites, virulence of the strain, and the *Leishmania* species involved.

iNKT cells produce multiple cytokines which can control the outcome of infection, frequently in favor of the host. However, sometimes they may lead to an uncontrolled cytokine storm and sepsis. The response of iNKT cells to pathogens is short term, and is followed by a prolonged refractory period of unresponsiveness to reactivation. This represents a method to avoid chronic activation and cytokine production by iNKT cells, protecting the host against the adverse effects of their activation but potentially putting the host at risk for secondary infections (11).

iNKT cells also mediate anti-tumor immunity by direct recognition of tumor cells that express CD1d and via targeting CD1d found on cells within the tumor microenvironment (3, 5). α-GalCers, a family of potent glycolipid agonists for iNKT cells, augment a wide variety of immune responses in vaccination against infections and can control tumor progression (1, 13).

Pro-inflammatory type II NKT cells are involved in the development of small vessel vasculitis in rats (6). In systemic lupus erythematosus (SLE), the quantity and quality of iNKT cells show marked defects. NKT cells affect the proportion of T-helper cells and the production of autoreactive antibodies as the disease progresses (14).

NKT cells are enriched in the liver. Although controversial, some studies have suggested that they have a potential role in hepatitis B virus and hepatitis C virus infections, autoimmune liver diseases, alcoholic liver disease, non-alcoholic fatty liver disease, and hepatocellular carcinoma (15–17). These differences may be due to the dynamic alterations of these cells during the progression of liver disease, which is caused by changes in their cellular subsets, cytokine responses, and intercellular crosstalk between NKT and CD1d-expressing cells or bystander cells (18).

## The Potential Role of NKT Cells in Immune Tolerance

A potential role for NKT lymphocytes in tolerance induction was shown under several pro-inflammatory settings including in animal models of immune-mediated hepatitis (19), colitis (20), diabetes, fatty liver disease-related inflammation (21–24), aortic valve disease (25), and cholangitis (26).

Compounds produced by sphingomyelin-ceramide-glycosphingolipid pathways have been studied as potential secondary messenger molecules. Some evidence suggested that they may act via promotion of NKT cells in settings of liver disorders and insulin resistance (27). Profiling of circulating phospholipids identified portal contributions to diabetes and a non-alcoholic steatohepatitis (NASH) signature in obesity (28). Portal and systemic phospholipid profiling revealed a NASH signature in morbid obesity (28). Increased concentrations of several glycerophosphocholines (PC), glycerophosphoethanolamines (PE), glycerophosphoinositols (PI), glycerophosphoglycerols (PG), lyso-glycerophosphocholines (LPC), and ceramides (Cer) were detected in the systemic circulation of NASH subjects (28). A beneficial effect was recently shown in humans with diabetes and NASH, as established by a liver biopsy, who were treated with β-glucosylceramide (GC) for 40 weeks (29). Oral administration of GC decreased the hepatic fat content measured by MRI in patients in the GC-treatment group compared to those in the placebo group. HbA1C was also reduced in patients treated with GC. GC treatment was associated with a milder decrease in the high-density lipoprotein serum levels. Beneficial effects were associated with a reduction in NKT cell subsets of lymphocytes (29).

Type II NKT cells that recognize the type II collagen peptide act as anti-inflammatory cells in different inflammation-induction models (6). A subset of type II NKT cells reactive to self-glycolipid sulfatides induces a dominant immune regulatory pathway that controls inflammation in autoimmunity as well as anti-cancer immunity (4).

CD4+ T cells from patients with SLE exhibit an altered outline of lipid raft-associated glycosphingolipids (GSLs). Increased levels of lactosylceramide, globotriaosylceramide (Gb3), and monosialotetrahexosylganglioside (GM1) were also noted. Elevated GSLs levels were described with increased expression of liver X receptor beta (LXRbeta), a nuclear receptor that controls cellular lipid metabolism and trafficking which also affects immune responses. Inhibition of GSL biosynthesis with an inhibitor (N-butyldeoxynojirimycin) which normalizes GSL metabolism, corrected CD4+ T-cell signaling and functional defects. It also decreased anti-dsDNA antibody production by autologous B cells in SLE patients (30).

## Can They Live Together?

As shown above, NKT cell subsets are of relevance for the maintenance of both pro-inflammatory and tolerance induction. Following activation, NKT cells rapidly secrete pro-inflammatory or anti-inflammatory cytokines, thereby determining the milieu for subsequent immunity or tolerance. GSLs were suggested as a mechanism for generating a new beneficial immune steady state in various immune environments. A beneficial tolerogenic effect on the immune system was shown for GC in both animals and humans (24, 31), while it was also shown to increase the potency of vaccination (32). A suggested effect on lipid rafts and downstream signaling pathway were shown (25, 33). That means that the same ligand or different ligands can correct the problem by targeting different or the same subset of cells. Thus, this can be viewed as a two-faced system for keeping the immune balance in opposing settings. This dichotomy was attributed to two different subsets of type I and type II NKT cells, which have different modes of antigen recognition and have opposing roles in inflammatory diseases (34). To determine whether GC can alter NKT function in opposing directions, it was tested in two immune-opposing models of colitis hepatocellular carcinoma (HCC) (35). Administration of GC led to alleviation of colitis and to suppression of HCC. The beneficial effects were associated with an opposite immunological impact in the two models with opposing effects on the peripheral to intrahepatic CD4:CD8 and NKT lymphocyte ratios. A similar beneficial effect of GC treatment on two immune-opposing models of graft vs. host disease was also shown (36). These studies demonstrate that GC can alleviate immunologically incongruous disorders and may be associated with “fine tuning” of immune responses, by altering the plasticity of NKT lymphocytes (35, 37).

These functional differences can be explained by the distinct antigen specificity exhibited among different subsets of NKT cells, the use of different ligands, environmental factors, targeting of diverse members of CD1d-restricted T cells including type I and type II NKT cells, and other non-invariant CD1d-restricted cell subsets; formation of stimulatory CD1d/antigen complexes; modes of TCR-mediated antigen recognition; and mechanisms of their activation. These factors underlie the functional differences in the diverse immune settings of infectious, inflammatory, and malignant diseases (8).

## Immune Changes in Patients With Gaucher Disease

Gaucher disease (GD) is an autosomal recessive deficiency of beta-glucocerebrosidase (GCase) (38). It is caused by mutations in the GBA1 gene encoding a lysosomal enzyme. GBA1 mutations drive the accumulation of GC in multiple innate and adaptive immune cells in the spleen, as well as in the liver, lungs, and bone marrow, often leading to chronic inflammation (39). These patients often present with alterations in cellular and humoral immunity. Alteration in CD4 and CD8 T-cell numbers results in a lower CD4/CD8 ratio and an increase in T-cell activation, along with decreased CD3+/CD4+ helper and increased CD3+CD8+ suppressor T lymphocytes, as well as a reduced CD4/CD8 cell ratio (40).

A decrease in B cell levels and an increase in NK and NKT cell levels were noted in patients with GD (40). Other studies showed that GD patients display decreased numbers of NK cells, gammadelta 2 T cells, and increased frequency of memory CD4(+)CD45RO(+) T cells (41). Numbers of myeloid dendritic cells (mDC) and plasmacytoid dendritic cells (pDC) were also decreased. pDC exhibited a decrease in IFNalpha production after TLR9 stimulation (41). These patients have impaired T-helper lymphocytes and a constitutive TH1 direction pattern of activation of both CD4+ and CD8+ cells, associated with a significant decrease in Tregs (42). They show increased IFNγ-producing CD4+ and CD8+ T cells and a decrease in CD4+CD25(dim) cells of CD4+CD25(high) T lymphocytes and CD4+CD25(high)FOXP3+ Tregs (42). Enzyme replacement therapy (ERT) may reverse some of these immune abnormalities (40).

Treatment of GluCerase-deficient monocytes from patients with GD or monocytes from healthy subjects with conduritol-B-epoxide (CBE), an irreversible inhibitor of GluCerase activity, induced production of CD1d and increased surface expression of major histocompatibility complex (MHC)-class II (43). A decrease in MHC-class II expression was seen in GD patients under enzyme replacement therapy (ERT), which positively correlated with chitotriosidase activity, a marker of inflamed macrophages. Retinoic acid (RA) and CBE upregulated CD1d expression induced by THP-1 cells. Treated THP-1 cells were more stimulatory for CD4(+) than for CD8(+) cells. Addition of α-GalCer expanded the iNKT, lineage, while adding isoglobotrihexosylceramide (iGb3), a potential physiological CD1d ligand, augmented the percentage of CD4 cells but did not expand the proportion of iNKT cells (44).

Chronic inflammation and B-cell activation is observed in GD. Beta-glucosylceramide 22:0 (betaGL1-22) and glucosylsphingosine (LGL1) accumulate in GD and are recognized by a distinct subset of CD1d-restricted type II NKT cells (45). In contrast to type I NKT cells, betaGL1-22- and LGL1-specific NKT cells express the T-follicular helper (TFH) phenotype. Injection of these lipids was found to increase lipid-specific type II NKT cells and germinal center B cells, hypergammaglobulinemia, and production of anti-lipid antibodies. The frequency of these cells is correlated with disease activity and therapeutic response (45). Following therapy, CD21(low) cells were reduced, and a subsequent increase in CD21(Hi) B lymphocytes occurred, indicating improved B-cell maturation. Class-switching and memory B cell defects, which were noted before treatment, were normalized (46).

Activation of complement C5a and C5a receptor 1 (C5aR1) controls GC accumulation. It was shown to regulate and the inflammatory response in GD. Local and systemic complement activation occurred in GCase-deficient mice or after pharmacological inhibition of GCase, associated with GC storage, tissue inflammation, and secretion of pro-inflammatory. Mice deficient in both GCase and C5aR1, or wild-type mice in which GCase and C5aR were pharmacologically inhibited, were protected, while GCase-inhibited mice died. GCase deficiency is associated with formation of complement-activating GC-specific IgG autoantibodies, activating complement and producing C5a. C5aR1 activation controls UDP-glucose ceramide glucosyltransferase production, tipping the balance between GC formation and degradation. The extensive GC storage prompts complement-activating IgG autoantibodies to the production of C5a and C5aR1 activation. This process is associated with a cycle of cellular GC accumulation and activation of innate and adaptive immune cells (39).

Macrophage inducible C-type lectin (Mincle) is an activating C-type lectin receptor that senses damaged cells. Mincle identifies glycolipid ligands on pathogens. GC was shown to be an endogenous ligand for Mincle and shows immunostimulatory activity (47). GC activated myeloid cells and induced inflammatory cytokines that were abrogated in Mincle-deficient cells. The inflammation was ameliorated in a Mincle-deficient background. A dynamic balance enables myeloid cells to respond rapidly when damage or stress occurs. Continuous recognition of a low-level ligand, such as GC, counterbalances inhibitory receptors that continuously recognize self (47).

Ineffective T-cell control and dysregulation of humoral immunity may explain the chronic inflammatory reaction and the increased incidence of lymphoid malignancies reported among patients with GD (41, 45). The impact of CD1d upregulation remains uncertain, and it has been proposed that MHC class II upregulation may be associated with inflammation in these patients (44). Conflicting results from pre-clinical and some clinical studies using ERTs and substrate-reduction therapies (SRT) show that both may be associated with inflammation, increased risk of cancer, and Parkinson's disease. There have been reports that some patients on ERT have developed type 2 diabetes or metabolic syndrome, malignancies, and central nervous system disorders. It was suggested that increased levels of GC might provide an evolutionary advantage for patients with GD, supporting the treatment of symptomatic patients with mild/moderate GD with low-dose ERT and re-evaluating the use of ERT in asymptomatic patients (48, 49).

For diabetes, an increase in GM3 levels *in vitro* alters the insulin receptor and may underlie insulin resistance. ERT corrects the insulin resistance defect in cultured adipocytes isolated from obese individuals and reduces macrophage numbers and chemoattractant that cause inflammation. Patients with GD have increased insulin levels and increased hepatic glucose production. Insulin resistance is present in non-overweight patients with GD who are treated with ERT (50). Notably, in untreated GD, the prevalence of overweight individuals was found to be lower than that in the general population. Long-term treatment with ERT prompts a higher than average weight gain and an increase in the prevalence of type II diabetes (51).

Much of the controversy can be attributed to the fact that many of the results from animal models are not applicable to human disease. *In vitro* data may therefore lack relevance *in vivo*. Moreover, the role of GC intracellularly may be different than when looking at serum levels of extracellular GC. Furthermore, many studies ignore the effect on other upstream and downstream GSLs. Dosages of ERT and SRT and splenectomy treatment may also explain some of the discrepancies between studies. Multiple host factors such as age, time of starting ERT/SRT therapies, and the presence of associated diseases, as well as the relatively small number of patients included in most studies, may contribute to the differences in results obtained in the different studies.

## Randomness in Biological Processes

Randomness is part of real-life biological processes and is being explored using complexity theory, chaos theory, and non-linear mathematics. It is suggested that biological sequences must resemble physics in generating order from disorder. This contrasts with Schrodinger's idea of biology generating order from a molecular-level order, which underlies much of our understanding of molecular biology (52).

Classical homeostasis implies that healthy systems aim at reducing variability and maintain steadiness. However, in many systems, variability is part of the normal physiological pattern (53). Healthy biological systems are dynamic networks of interactions that show randomness and fluctuations (54–57). Under resting conditions, healthy systems display highly irregular, complex dynamics (58). Non-linear dynamics suggests that a disease is associated with loss of complexity and inability to restore the original complex physiological state (52, 59–62).

Normal heart rate variability (HRV), a measure of the beat-to-beat variation in heart rate, is characterized by fractional noise. HRV alterations can be used to predict sudden death, mortality in patients with myocardial infarction, heart failure, and for the prognostic assessment of elderly patients (53, 63). The fractal organization has been observed in the physiologic breathing cycle dynamics. A decrease in the complexity of respiration occurs with aging (64). Similar variability was described for brain function, which persists despite the removal of all temporal noise from the stimulus (65). A reduction in complexity is associated with arthritis, stroke, Parkinson's disease, and Alzheimer's disease (66–68). The secretion of many hormones oscillates in a fractal-like manner and follows the rules of chaos theory (69). Immune cells are less capable of recognizing antigens upon aging due to a reduction in the complexity of their sub-components. This effect may result in autoimmune diseases, chronic inflammation, cancer, and infections (70).

The randomness concept in biology also challenges the traditional pharmacological regimes of prescribing medications based on regular administration. It suggests that, for maximum benefit, medications should be administered at irregular intervals and at continually changing dosages (55, 71, 72).

Traditional immunology looks at altering the immune system into opposing directions or at regulatory cell plasticity, as resulting from different types of activation of the same cell, leading to different cytokine secretion patterns. Alternatively, skewing the immune system may be a result of activation of a different subset of cells. Indeed, much of the dichotomy described for NKT cells is explained by analyzing different subsets of lymphocytes, or different ligands activating the same subset under different settings. While these may be of relevance in laboratory settings, they lack complete accuracy for the human biological system. Lack of consistency in the response to ligands for NKT cells in clinical trials is attributed to multiple parameters and the diversity of the patient population. However, opposing effects under different settings and the lack of consistent effects when targeting NKT cells may also be attributed to inherent randomness in the function of these cells(73–75).

It was recently suggested that randomness could increase the efficacy of biological systems. This theory applies some concepts from quantum physics into biology, while viewing biological systems as multifactorial, which makes them different from any other system in physics, whose parameters are easier to control (52, 62). Variations in genotypic and phenotypic parameters make patient-tailored processes critical. It implies that types of randomness may not be identical in all patients and that they are not expected to produce the same effect in all cases. In fact, personalized randomness-based treatments were suggested to be designed by selecting one or more parameters in a custom-made, random-dependent manner. This methodology is viewed as an advanced level of randomness intended for improving the efficacy of biological systems in a personalized way.

## Applying the Randomness Concept to GD Treatment

**Figure 1:**
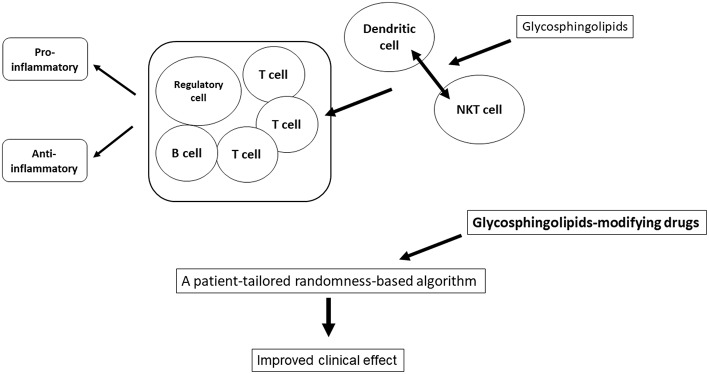
Applying the randomness to treatment. A balance between various glycosphingolipids is essential for immune homeostasis. Glycosphingolipids promote an immune balance by changing different pathways of the immune system in opposing environments. Generation of tailored-randomness can be used for improving the consistency of response to immunotherapies.

A balance between various glycosphingolipids is essential for immune homeostasis. Glycosphingolipids promote an immune balance by changing different pathways of the immune system in opposing environments. The effect of β-glycosphingolipids may be associated with promoting the DC-NKT interaction, regulatory T cells, and alteration of intracellular signaling. Generation of tailored-randomness can be used for improving the consistency of response to immunotherapies, such as when using ligands for NKT cells (Figure 1). These algorithms may help improve the effectiveness of ERT/SRT treatment by providing therapies in a random manner in regards to administration time and dosage, allowing for patient-tailored delivery that is based on pre-defined positive endpoints.

## Author Contributions

The author confirms being the sole contributor of this work and has approved it for publication.

### Conflict of Interest Statement

YI is a consultant for Teva; ENZO; Protalix; Betalin Therapeutics; Immuron; SciM; Natural Shield; founder of Oberon Sciences; Tiziana Pharma; Plantylight; Exalenz Bioscience. YI is the founder of Oberon Sciences.

## Abbreviations

NKT cells: Natural Killer T cells
iNKT cells: Invariant NKT cells
α-GalCer: alpha-galactosylceramide
GD: Gaucher disease
GCase: glucocerebrosidase
GC: β-glucosylceramide
ERT: enzyme replacement therapy
mDC: myeloid dendritic cells
pDC: plasmacytoid dendritic cells
Tregs: regulatory T cells
CBE: conduritol-B-epoxide
MHC: major histocompatibility complex
iGb3: exogenous isoglobotrihexosylceramide
TFH: T-follicular helper
APCs: antigen-presenting cells
HCC: hepatocellular carcinoma
Mincle: Macrophage inducible C-type lectin
NASH: non-alcoholic steatohepatitis
PC: glycerophosphocholines
PE: glycerophosphoethanolamines
PI: glycerophosphoinositols
PG: glycerophosphoglycerols
LPC: lyso-glycerophosphocholines
Cer: ceramides
GSLs: glycosphingolipids
LXRbeta: liver X receptor beta
SRT: substrate reduction therapies
HRV: heart rate variability.
